# Angina Bullosa Hemorrhagica

**DOI:** 10.31662/jmaj.2022-0108

**Published:** 2022-12-19

**Authors:** Yasuhiro Kano

**Affiliations:** 1 Department of General Internal Medicine, Tokyo Metropolitan Tama Medical Center, Tokyo, Japan

**Keywords:** angina bullosa hemorrhagica, diabetes mellitus, traumatic oral hemophlyctenosis, recurrent oral hemophlyctenosis

## Abstract

Angina bullosa hemorrhagica (ABH) is an underrecognized, benign condition of the oral mucosa. A 26-year-old female patient with type 2 diabetes mellitus presented with sudden-onset painless blood blisters on her soft palate. ABH was clinically diagnosed based on the clinical presentation and spontaneously resolved. Some medical conditions, including diabetes mellitus, hypertension, and inhaled steroids, can be a risk factor of ABH. Clinicians should be aware of ABH and consider the possibility of an associated underlying condition.

## Introduction

Angina bullosa hemorrhagica (ABH) is a benign, self-limited condition characterized by the sudden onset of painless blood-filled blisters in the oral cavity. ABH mainly affects the soft palate predominantly in middle-aged to elderly adults ^(1), (2), (3)^. Although ABH is underrecognized, clinicians should be familiar with it because of its potential association with an underlying condition, such as diabetes mellitus, hypertension, or inhaled steroids ^(1), (4), (5), (6)^.

Most previous cases of ABH have been reported by dentists or dermatologists. Thus, comparatively few physicians may recognize the disease despite its possible association with common medical conditions. We report herein a case of ABH in a patient in her 20s, which was possibly caused by preexisting diabetes mellitus.

## Case Report

**Figure 1.**
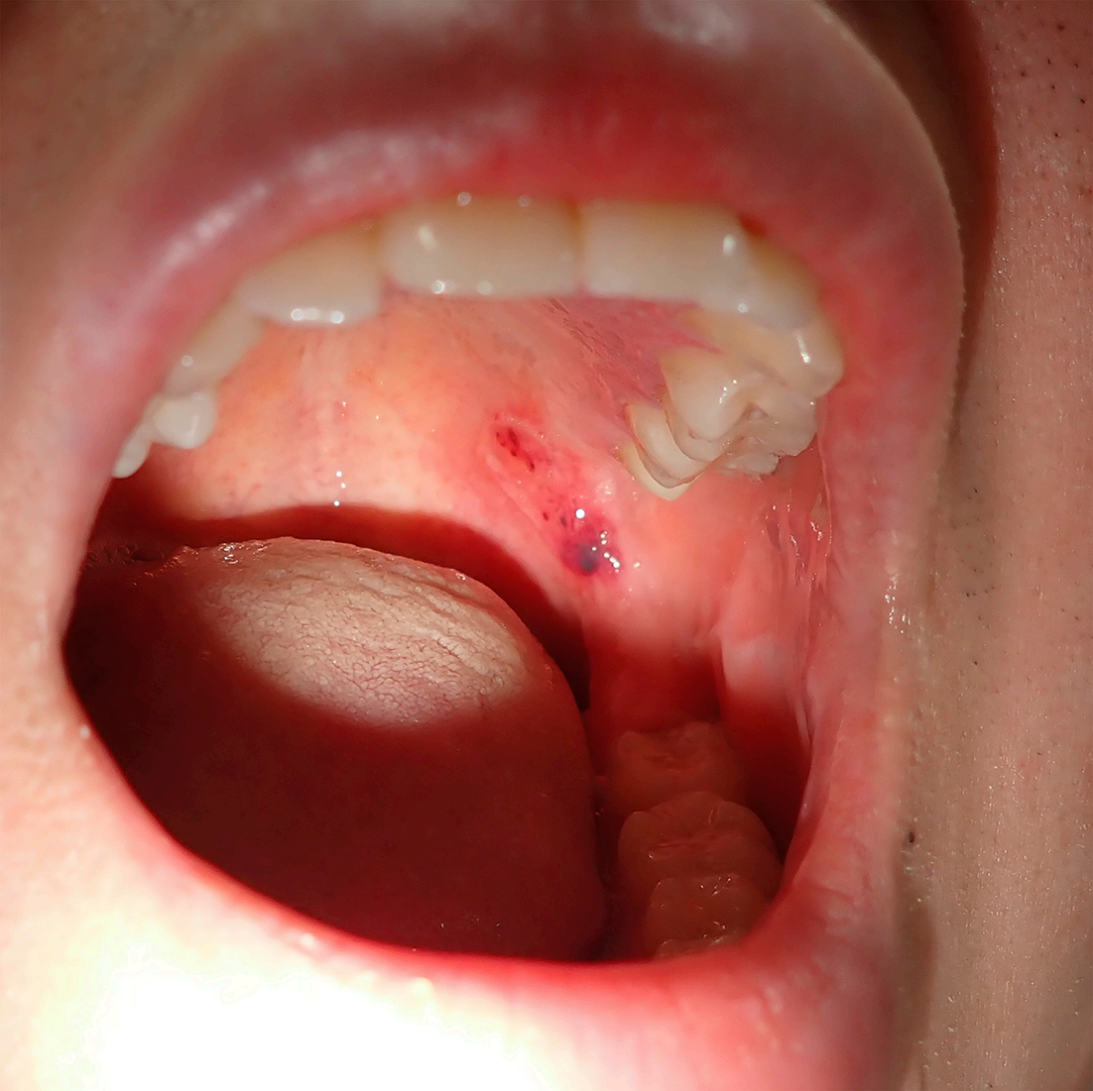
One blood blister and one postbullous erosion on the soft palate.

A 26-year-old female Japanese office worker with type 2 diabetes mellitus presented with painless blood blisters in her oral cavity. Five years prior to her current presentation, she was diagnosed with type 2 diabetes mellitus, for which she was receiving glimepiride, pioglitazone, and tofogliflozin. Two blood-filled blisters had suddenly developed during a meal, and one of them burst soon thereafter. She had experienced similar episodes during meals over the previous 2 years. Physical examination revealed one blood blister and one postbullous erosion on her soft palate (Figure 1). Laboratory tests revealed a normal platelet count (231 × 10^9^/L), no coagulopathy, normal prothrombin time and international normalized ratio (0.91), and normal liver enzymes, including aspartate aminotransferase (17 IU/L) and alanine aminotransferase (24 IU/L). Her glycated hemoglobin (HbA1c) and random plasma glucose were 5.6% and 87 mg/dL, respectively. ABH was clinically diagnosed. The patient’s lesions healed within 1 week without treatment.

## Discussion

The term ABH was first introduced by Badham in 1967 to describe recurrent blisters with a hematic content in the oral cavity that could not be attributed to any hematological, dermatological, or systemic disease ^(7)^. ABH is a self-limiting condition characterized by the sudden onset of blood-filled blisters in the oral cavity, which rupture and heal spontaneously. The pathogenesis remains unclear, but minor trauma and vascular fragility may play a role ^(1)^. ABH is more common in middle-aged to elderly adults, with the highest incidence among those aged >50 years and no apparent difference in terms of sex ^(1), (2), (3)^. No study has thus far investigated the incidence and prevalence of ABH although it is thought to be underdiagnosed because it may be frequently mistaken for another illness and resolves rapidly and spontaneously ^(8)^.

ABH typically develops during meals, and the soft palate is most commonly affected ^(9)^. The lesions are often painless or asymptomatic but may cause a choking sensation (hence the term, angina); they seldom cause dyspnea or respiratory distress ^(10)^. The differential diagnosis for ABH includes mucous membrane pemphigoid, oral amyloidosis, pemphigus vulgaris, bullous pemphigoid, acquired epidermolysis bullosa, linear IgA dermatosis, herpetiform dermatitis, and bullous lichen planus ^(3), (8), (11)^. A complete blood count and coagulation panel should always be performed to rule out systemic hematological pathologies, including leukemia, thrombocytopenia, and von Willebrand disease, because they can present with lesions similar to those of ABH ^(2)^.

Importantly, a possible association has been reported with inhaled steroids and certain underlying systemic conditions, including diabetes mellitus and hypertension ^(1), (4), (5), (6)^. Grinspan et al. reported that 44.4% cases of ABH had diabetes mellitus, hyperglycemia, and/or a family history of diabetes ^(1)^. The present patient also had type 2 diabetes, which may be why ABH developed despite her relatively young age.

The prognosis of ABH is benign, and no treatment is usually needed ^(1), (8)^. The lesions show a favorable evolution without scarring after a few days ^(12)^. Therefore, ABH management consists mainly of reassuring the patient and modifying the underlying risk factors although symptomatic therapy, including analgesics for pain, may be considered. Some studies recommend using a mouth wash containing 0.12%-0.2% chlorhexidine gluconate to prevent a secondary infection ^(1)^.

Most previous cases of ABH have been reported by dentists or dermatologists. Thus, comparatively fewer physicians may recognize the disease despite its possible association with some common medical conditions. Clinicians should be aware of ABH as a possible phenotype of a common chronic condition, such as diabetes mellitus or hypertension, especially if it occurs in a young patient.

## Conclusion

ABH is a benign, self-limiting condition that can usually be diagnosed confidently on the basis of history taking and physical examination. Clinicians should be aware of this entity and reassure patients of its benign prognosis while considering the possibility of an associated underlying condition, such as diabetes mellitus or hypertension.

## Article Information

### Conflicts of Interest

None

### Acknowledgement

The author thanks James R. Valera for assistance with editing the manuscript.

### Author Contributions

Yasuhiro Kano contributed in patient care, designing and conducting the study, and writing the manuscript.

### Patient Consent

Consent to publish the details of the present case was obtained from the patient.
